# Crystal and Magnetic Structure Transitions in BiMnO_3+δ_ Ceramics Driven by Cation Vacancies and Temperature

**DOI:** 10.3390/ma14195805

**Published:** 2021-10-04

**Authors:** Dmitry V. Karpinsky, Maxim V. Silibin, Dmitry V. Zhaludkevich, Siarhei I. Latushka, Vadim V. Sikolenko, Daniel M. Többens, Denis Sheptyakov, Vladimir A. Khomchenko, Alexei A. Belik

**Affiliations:** 1 Laboratory of Technology and Physics of Crystals Growth, Scientific-Practical Materials Research Centre of NAS of Belarus, 220072 Minsk, Belarus; geludkevichdima@mail.ru (D.V.Z.); latushkasi@gmail.com (S.I.L.); 2 Institute for Advanced Materials and Technologies, National Research University of Electronic Technology “MIET”, 124498 Zelenograd, Russia; sil_m@mail.ru (M.V.S.); sikolen@jinr.ru (V.V.S.); 3 Department of Materials Science and Physico-Chemistry of Materials, South Ural State University, av. Lenina, 76, 454080 Chelyabinsk, Russia; 4 Scientific-Manufacturing Complex “Technological Centre”, 124498 Zelenograd, Russia; 5 Institute for Bionic Technologies and Engineering, I.M. Sechenov First Moscow State Medical University, 119991 Moscow, Russia; 6 Frank Laboratory of Neutron Physics, Joint Institute for Nuclear Research, 141980 Dubna, Russia; 7 Institute of Applied Geosciences, Karlsruhe Institute of Technology, 76131 Karlsruhe, Germany; 8 Department Structure and Dynamics of Energy Materials, Helmholtz-Zentrum Berlin für Materialien und Energie, 14109 Berlin, Germany; daniel.toebbens@helmholtz-berlin.de; 9 Laboratory for Neutron Scattering and Imaging, Paul Scherrer Institut, 5232 Villigen, Switzerland; denis.cheptiakov@psi.ch; 10 CFisUC, Department of Physics, University of Coimbra, 3004-516 Coimbra, Portugal; uladzimir@uc.pt; 11 International Center for Materials Nanoarchitectonics (WPI-MANA), National Institute for Materials Science (NIMS), Namiki 1-1, Tsukuba, Ibaraki 305-0044, Japan; alexei.belik@nims.go.jp

**Keywords:** multiferroics, crystal and magnetic structure, phase transitions, orbital ordering, magnetometry, synchrotron and neutron diffraction

## Abstract

The crystal structure of BiMnO_3+δ_ ceramics has been studied as a function of nominal oxygen excess and temperature using synchrotron and neutron powder diffraction, magnetometry and differential scanning calorimetry. Increase in oxygen excess leads to the structural transformations from the monoclinic structure (*C2/c*) to another monoclinic (*P2*_1_*/c*), and then to the orthorhombic (*Pnma*) structure through the two-phase regions. The sequence of the structural transformations is accompanied by a modification of the orbital ordering followed by its disruption. Modification of the orbital order leads to a rearrangement of the magnetic structure of the compounds from the long-range ferromagnetic to a mixed magnetic state with antiferromagnetic clusters coexistent in a ferromagnetic matrix followed by a frustration of the long-range magnetic order. Temperature increase causes the structural transition to the nonpolar orthorhombic phase regardless of the structural state at room temperature; the orbital order is destroyed in compounds BiMnO_3+δ_ (δ ≤ 0.14) at temperatures above 470 °C.

## 1. Introduction

In the last two decades, complex oxides of transition metals possessing both magnetic and electric dipole orders (so called multiferroics) have attracted increased interest of researchers [1,2]. The most studied single-phase multiferroic is bismuth ferrite, which has high transition temperatures to magnetic (650 K) and ferroelectric (1100 K) phases [1,3]. Bismuth manganite (BiMnO_3_) is also a well-known magnetoelectric material with perovskite structure with transition temperature to the magnetically ordered state of T_C_ ~ 102 K [4]. The ferromagnetic (FM) order in BiMnO_3_ is caused by the presence of orbital ordering, which is destroyed at a temperature of ~475 K; while the type of structural distortion does not change, the crystal structure remains to be monoclinic; at temperatures above T ~ 770 K, the crystal structure changes to orthorhombic (space group (SG) *Pnma*) [5]. The magnetic structure of BiMnO_3_ is determined by positive exchange interactions formed between Mn^3+^ ions, while the type of the exchange interactions strongly depends on the mutual orientation of the 3 d orbitals of Mn ions as well as the geometry of the Mn-O-Mn chemical bonds [6].

It is known that the crystal structure and magnetic properties of BiMnO_3_-based compounds can be significantly modified via chemical doping [7,8]. Magnetic properties of BiMnO_3_-based compounds can also be controllably changed via doping on the A- and/or B- perovskite positions [9,10]. It is known that isovalent substitution in the A-position with rare-earth elements causes a stabilization of long-range antiferromagnetic (AFM) order accompanied by an increase in the magnetic transition temperature [11], whereas the nominal oxidation state of the Mn ions remains unchanged. Chemical substitution of Mn ions for other transition-metal ions also causes a disruption of ferromagnetic order [4,12], thus confirming a dominant role of the orbital order in magnetic properties of the BiMnO_3_-based compounds [13].

The chemical doping schemes assume an influence of the dopant ions on the crystal structure and electronic configuration of the B-site cations and their chemical bond character, thus hampering an analysis of the exchange interactions formed between manganese ions. On the other hand, a self-doping approach [14] can be used as an entirely internal stimulus which causes a modification of the crystal structure and thus changes the magnetic state of the compounds, thus facilitating an understanding of the structure-properties relationship. In the present study, we have investigated the crystal structure of ceramics BiMnO_3+δ_ depending on the intrinsic (cation vacancies) and extrinsic (temperature) stimuli—the factors affecting magnetic state of the compounds. The obtained results have allowed us to clarify a correlation between the cation content, crystal structure and orbital ordering in the ceramic compounds BiMnO_3+δ_ at different temperatures, thus showing that cation vacancies can be used to control orbital order and thus magnetic properties of the manganites.

## 2. Experimental

Ceramic compounds BiMnO_3+δ_ with nominal oxygen excess δ = 0.02, 0.08, 0.14 were prepared from simple oxides Bi_2_O_3_ (99.9999%, Rare Metallic Co. Ltd.), MnO_2_ (99.997%, Alfa Aesar), and Mn_2_O_3_ taken in accordance with the chemical formulas Bi_0.993_Mn_0.993_O_3_, Bi_0.974_Mn_0.974_O_3_ and Bi_0.955_Mn_0.955_O_3_ respectively. Mn_2_O_3_ was prepared from MnO_2_ by heating in air at 923 K in 5 h. The oxygen content and phase purity of MnO_2_ were confirmed by X-ray powder diffraction and thermogravimetric analysis (TGA) before its use. The synthesis was performed using a belt-type high-pressure apparatus at a pressure of 6 GPa and temperature of about 1600 K for 40 min in sealed Pt capsules. After synthesis, the pressure was slowly released, and the samples were quenched at room temperature. Chemical composition of the compounds and the cation ratio was confirmed by electron probe microanalysis and density analysis as described in a previous study [15] as well as precise structural data presented below. The crystal and magnetic structure of the compounds were analyzed using X-ray diffraction data obtained with a PanAlytical X’pert Pro diffractometer (Panalytical B.V., Almelo, Netherlands), synchrotron powder diffraction (SPD) data obtained at the KMC-2 beamline (BESSY-II electron storage ring [16]), and neutron diffraction measurements performed with the HRPT diffractometer (PSI) [17]. Temperature dependent SPD measurements were run under ambient atmosphere in the range 10° < 2ϴ < 100° and temperatures 290–970 K with a step of 0.014° at λ ~ 1.77 Å; neutron diffraction data were recorded in the temperature range 290–750 K (λ ~1.494 Å). The X-ray and neutron diffraction data were analyzed by the Rietveld method using the FullProf software [18,19]. Magnetization measurements were performed using Cryogen Free Measurement System (CFMS) (Cryogenic ltd, London, UK). Differential scanning calorimetry (DSC) measurements were carried out with a Phoenix 204 F1 setup (Netzsch GmbH, Selb, Germany) in nitrogen flow at a heating and cooling rate of 10 °C per min in the range 300–700 K (nitrogen is considered as a neutral purge gas having high heat conductivity in the mentioned temperature range).

## 3. Results and Discussion

Analysis of the laboratory and synchrotron X-ray diffraction patterns of the compounds BiMnO_3+δ_ confirms the changes of crystal structures from the monoclinic structure (*C2/c*) to another monoclinic (*P2*_1_*/c*) and then to the orthorhombic structure (*Pnma*) with nominal increase in the oxygen content which is consistent with available data [15]. The mentioned sequence of the structural transformations is accompanied by a gradual decrease in the unit cell volume, while the crystal symmetry of the compounds changes nonmonotonously. Along with the information about the structural transformation, the diffraction measurements have also provided detailed information about the structural parameters, ionic coordinates and occupations. The occupation values calculated for the bismuth and manganese ions confirm nearly equal amount of the cation vacancies of about 3% and 5% for the compounds with nominal oxygen excess δ = 0.08 and 0.14 which correspond to their chemical formulas. Limited accuracy of the diffraction data could not exactly approve the chemical composition of the compound BiMnO_3.02_, while the reliability factors can be notably improved assuming a small deficit in the cation occupations during the refinement procedure. Further decrease in the cation/anion ratio specific to the BiMnO_3.08_ sample leads to a stabilization of the monoclinic structure having lower symmetry, viz. *P2*_1_*/c* (SG #14) as compared to the stoichiometric compound—*C2/c* (SG #15)—while further decrease in the cation content leads to a stabilization of the nonpolar orthorhombic structure *Pnma* (SG #62). 

**Figure 1:**
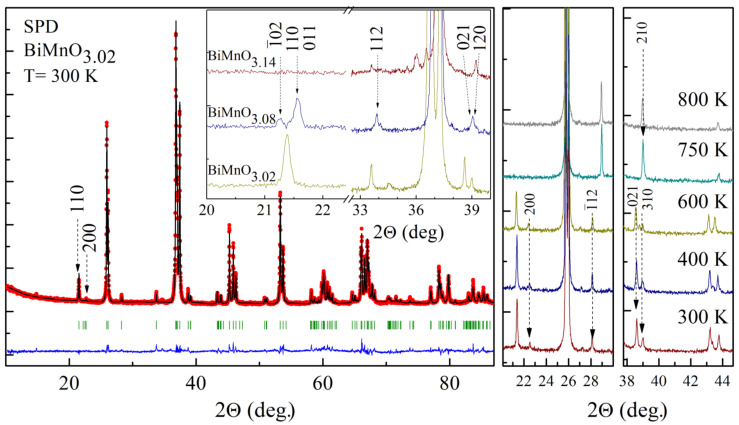
SPD pattern of the compound BiMnO_3.02_ refined using the space group *C2/c*. Inset shows the reflections specific to the compounds BiMnO_3+δ_ (δ = 0.02, 0.08, 0.14). Images on the right show the temperature evolution of the selected SPD reflections of the compound BiMnO_3.02_.

It should be noted that the space group *P2*_1_*/c* is the only common maximal subgroup for both *C2/c* and *Pnma* space groups. The symmetry of the crystal structure of BiMnO_3.08_ is lower than that attributed to BiMnO_3.02_ as determined by the extinction rule (Figure 1) [20]. Indeed, the diffraction pattern obtained for BiMnO_3.08_ reveals a splitting of the reflection located at 2ϴ ≈ 21.7 deg., and the extra reflection appears at 2ϴ ≈ 21.4 deg. (indexed as $\bar{1}02$ in the space group *P2*_1_*/c* (Figure 1)), which points to the symmetry lowering. An appearance of the extra reflections associated with a lowering of the crystal symmetry is caused by an occupation of the new less-symmetrical positions of the manganese and oxygen ions over the lattice. The space group *P2*_1_*/c* (without centering) allows a splitting of the reflection 110, thus leading to an appearance of the satellite 011 forbidden in *C2/c*; the extra reflection $\bar{1}02$ is associated with Mn ions in the Wyckoff position *2c* (0; 0; ½) formed as a result of splitting of the Wyckoff position *4d* (¼; ¼; ½) specific for *C2/c* into *2c* (0;0; ½) and *2b* (½; 0; 0) positions of the space group *P2*_1_*/c* (Table 1). Further decrease in the amount of the cation content down to ~95.5% (BiMnO_3.14_) from the stoichiometric composition leads to a decrease in the unit cell volume resulting in a stabilization of orthorhombic symmetry (*Pnma*). The orthorhombic lattice is characterized by one structural position for Mn ions—*4b* (0; 0; ½) (Table 1) and two different positions occupied by oxygen ions—*4c* and *8d*.

**Figure 2:**
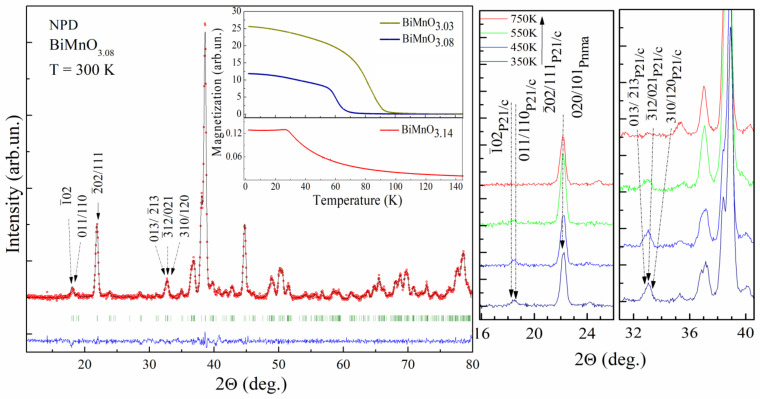
Refined NPD pattern of the compound BiMnO_3.08_ using the space group *P2*_1_*/c*. Inset shows M(T) magnetization curves for the compounds BiMnO_3+δ_ (δ = 0.02, 0.08, 0.14) measured in a field-cooled mode (H~100 Oe). Images on the right show the temperature evolution of selected NPD reflections of the compound BiMnO_3.08_.

Decrease in the cation content leads to a reduction of magnetization of the compounds as declared in the previous works [15,21] and confirmed by our neutron diffraction and magnetization data (Figure 2). The changes in the magnetic structure are mainly caused by two factors—modification in the orbital ordering of the manganese ions and a dilution of the magnetic sublattice by the cation vacancies. The first factor is determined by the geometry of the bond lengths Mn-O and angles Mn-O-Mn, the second factor is associated with a character of the vacancies distribution over the A- and B- sublattices of the perovskite structure. Both mentioned factors have different impact on the magnetic structure of the compounds.

A decrease in the cation content even up to ~ 5% (in case of the compound BiMnO_3.14_) should not necessarily lead to a suppression of the long-range magnetic order observed experimentally [15,21] (Figure 2), e.g., other manganites as LaMnO_3_ and LuMnO_3_, keep stable long-range magnetic structure up to 6–10% of the Mn vacancies [14,22,23,24].

**Figure 3:**
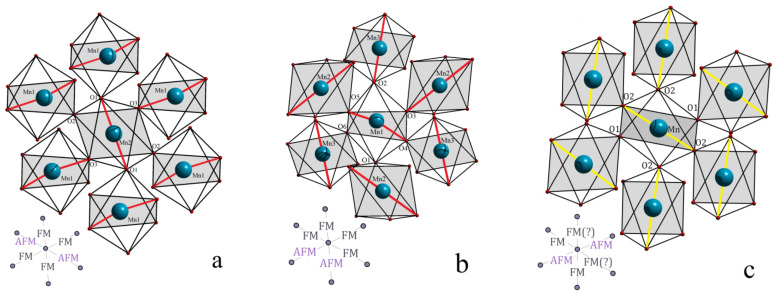
Schemes of the oxygen octahedra of the compounds BiMnO_3+δ_; (**a**) denotes the scheme specific to the compound with δ = 0.02; (**b**) —δ = 0.08; (**c**) —δ = 0.14); the *d_z_^2^* orbitals are highlighted in red and yellow colors (yellow color of the *d_z_^2^* orbitals in case of BiMnO_3.14_ compound denotes partial suppression of the orbital ordering which leads to an uncertainty in the type of the exchange interactions Mn-O(2)’-Mn, marked by “?”).

The orbital ordering and the magnetic state of the compounds BiMnO_3+δ_ are strongly dependent on the spatial orientation of the chemical bond lengths and thus the type of the exchange interaction between Mn ions which can be of negative or positive character. Careful analysis of the diffraction data has allowed us to clarify that in the compound BiMnO_3.02_, the manganese vacancies are mainly located in the Wyckoff position *4d* (¼; ¼; ½) occupied by Mn(2) ions, while the orbital ordering remains the same as it was observed in the stoichiometric compound [13]. Thus, in the compound BiMnO_3.02_ with crystal structure characterized by two independent positions of Mn ions, viz. *4d* (¼; ¼; ½) and *4e* (0; y; ¾), the exchange interactions Mn(1)-O(1)-Mn(2) and Mn(1)-O(3)-Mn(2) have positive character while that between Mn(1)-O(2)-Mn(2) is of negative character (Table 1, Figure 3). The positive character of the exchange interactions Mn(1)-O(1, 3)-Mn(2) is determined by the Goodenough–Kanamori rules [25,26], as the mentioned couplings are formed by the half-filled *d_z2_* orbital of Mn(1) ions and the empty *d_x2-y2_* orbital of Mn(2) ions; in turn, negative character of the exchange interactions Mn(1)-O(2)-Mn(2) is associated with a coupling between two empty *d_x2-y2_* orbitals.

Lowering of the structural symmetry observed for the compound BiMnO_3.08_ is caused by an ordering of the cation vacancies. It results in a presence of three different structural positions for the Mn ions. Based on the refined structural data, we conclude that in the compound BiMnO_3.08_, the manganese vacancies are mainly located in the Mn(2) position *2b* and partly in the Mn(1) position *4e*. It should be noted that the space groups *C2/c* and *P2*_1_*/c* follow the group–subgroup relation and the position *4d* in the SG *C2/c* is split into the positions *2b* and *2c* in the SG *P2*_1_*/c*. Modification of the crystal structure occurring in the BiMnO_3.08_ compound as compared to BiMnO_3.02_ results in a modification of the geometry of the orbital ordering. The orbital ordering specific to the BiMnO_3.08_ compound is characterized by four positive and two negative exchange interactions (Figure 3b, Mn(1) ion is located in the center). While in the BiMnO_3.02_ compound (Figure 3a) four positive exchange interactions form two pathways with long-range ordered ferromagnetic coupling, in the BiMnO_3.08_ compound, there is only one pathway associated with long-range ferromagnetic order; the next one has frustrated character where positive and negative interactions are alternated and the last pathway is characterized by negative interactions thus fostering a long-range antiferromagnetic order (Table 1, Figure 3b).

In the BiMnO_3.14_ compound, the orbital ordering is strongly suppressed as confirmed by the structural analysis, viz. lesser difference between the Mn-O bond lengths (Figure 3c, Table 1), wherein the manganese vacancies drastically frustrate positive superexchange interactions thus leading to a further decrease in the magnetization. It should also be noted that the charge neutrality in the nonstoichiometric compounds BiMnO_3+δ_ is achieved through the formation of Mn^4+^ ions [14]. To keep the electric neutrality in the BiMnO_3.14_ compound, the concentration of Mn^4+^ ions should be about 28%; a similar value is derived from the BVS (bond valence sum) parameters calculated during the structure refinement procedure. The estimated amount of the Mn ions in the 4+ oxidation state should dramatically affect the magnetic structure through a suppression of the orbital order in the compound as well as providing strongly positive exchange interactions Mn^4+-^O-Mn^3+^. Based on the available data, one can conclude that the magnetic properties of the BiMnO_3.14_ compound are mainly determined by partly suppressed orbital ordering as positive exchange interactions Mn^4+^-O-Mn^3+^ have only a local character and do not contribute to the formation of long-range ferromagnetic order [27].

Based on the obtained structural data, we conclude that the cation (manganese) vacancies and the character of their distribution over the lattice provide a change in the geometry of the orbital arrangement, thus causing a suppression of the ferromagnetic properties. One should also take into account a formation of Mn^4+^ ions that ensure the charge neutrality of the nonstoichiometric compounds, while even in the sample with the maximal amount of the cation vacancies (BiMnO_3.14_), the Mn^4+^ ions did not notably provide a revival of the ferromagnetic order.

### Temperature Dependent Structural Investigations

Analysis of the temperature-dependent synchrotron X-ray and neutron diffraction data has allowed us to clarify an evolution of the structural state and to evaluate the modification of the orbital ordering in the compounds having different structures at room temperature. Temperature-dependent structural data permitted an estimation of the impact of the cation vacancies on the temperature stability of the different structural states and orbital ordering which determines the magnetic structure and properties. 

**Figure 4:**
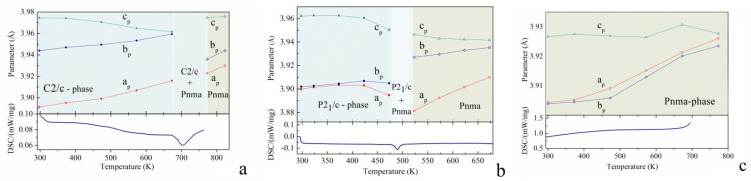
The unit cell parameters derived from the SPD and NPD data of the compounds BiMnO_3+δ_ with δ = 0.02, 0.08, 0.14 - (**a**–**c**), respectively. The DSC curves obtained for the respective compounds are presented at the bottom. The unit cell parameters are presented in normalized form, viz. a_p_ = a/√6, b_p_ = b/√2, c_p_ = c/√6·(for *C2/c* and *P2*_1_*/c* phases) and a_p_ = a/√2, b_p_ = b/4, c_p_ = c/(2√2)·(for *Pnma* phase).

In case of the BiMnO_3.02_ compound, a temperature increase leads to a gradual reduction in the structural distortion as confirmed by a decrease in the intensity of the reflections specific to the monoclinic structure, e.g., 110, −112, 112, 312 (Figure 1). In particular, the unit cell parameters gradually increase with temperature showing ~1% increment in the unit cell volume over the range 320–670 K associated with monotonous increase of the *a-* and *b-* parameters (Figure 4), whereas the *c-*parameter slowly decreases, thus leading to more symmetric structure. Temperature increase above 670 K leads to a drastic modification in the unit cell parameters associated with a structural transition to the nonpolar orthorhombic phase with *Pnma* symmetry. The temperature range of the orthorhombic phase stability is quite narrow (770–820 K); further temperature increase leads to a chemical decomposition of the sample as confirmed by the diffraction data and the results of the DSC measurements. It should be noted that the structural transition into the orthorhombic phase is accompanied by a decrease in the primitive cell volume of about 0.7% pointing at more dense packing of the oxygen octahedra in the lattice. Analysis of the chemical bond lengths allowed us to conclude that the orbital ordering found in the BiMnO_3.02_ compound is destroyed at temperatures above 500 K, the disruption occurring in the narrow temperature range of about 50 K. The data obtained show that the orbital ordering collapses simultaneously along three pathways in contrast to the concentration induced modification of the orbital ordering.

In the BiMnO_3.08_ compound, the structural phase transition to the nonpolar orthorhombic phase occurs at lower temperature as compared to the BiMnO_3.02_ compound. At a temperature of ~470 K, the structural state of the compound BiMnO_3.08_ is characterized by two coexisting phases—monoclinic *P2*_1_*/c* and the nonpolar orthorhombic with the space group *Pnma*. At temperatures above 500 K, the crystal structure of the compound BiMnO_3.08_ is single phase orthorhombic up to 670 K. Above this temperature, the compound begins to decompose. Whereas the difference in the cell volumes calculated for the monoclinic and orthorhombic phases (0.4%) is notably smaller as compared to the data obtained for the BiMnO_3.02_ compound, the diffraction measurements confirm the absence of any intermediate structural phase in this phase transition.

The structural parameters calculated for the BiMnO_3.08_ compound within the monoclinic phase demonstrate the tendency previously observed for the BiMnO_3.02_ compound with the temperature increase, assuming a collapse of the orbital ordering at a temperature of ~470 K. The compound BiMnO_3.14_ maintains the orthorhombic structure up to ~820 K, where the chemical decomposition starts. An evolution of the calculated lattice parameters points to a nonmonotonous change of the structural distortions. The derived chemical bond lengths indicate a complete disruption of the orbital order at temperatures above 470 K, which might also be indicated by a modification of the lattice parameters about this temperature (Figure 4).

## 4. Conclusions

Synchrotron X-ray and neutron diffraction measurements allowed clarification of the details of the evolution of the crystal and magnetic structures of the BiMnO_3+δ_ compounds as a function of temperature and the cation content. One can conclude that the structural stability of the monoclinic phases reduces with an increase in the number of the cation vacancies. The temperature of the structural transitions to the nonpolar orthorhombic phase reduces with an increase in the value of nominal oxygen excess; the orbital ordering specific to the compounds is destroyed at temperatures about 500 K. Increase in the number of the cation vacancies causes a rearrangement of the orbital order (which is accompanied by a change of the dominant superexchange interactions) followed by a disruption of the orbital ordering and removal of the long-range spin order.

## Figures and Tables

**Table 1:** Selected structural parameters of the BiMnO_3+δ_ compounds refined based on the synchrotron and neutron diffraction data.

Sample	SG	Mn-O-Mn/deg.	Magn. Order	Mn-O/Å
BiMnO_3.02_	*C2/c*	Mn(1)-O(1)-Mn(2) (148.05)	FM	Mn(1)-O(1): 1.955(7); Mn(1)-O(2): 2.037(5); Mn(1)-O(3): 2.076(4); Mn(2)-O(1): 2.125(8); Mn(2)-O(2): 2.081(5); Mn(2)-O(3): 1.899(7);
		Mn(1)-O(2)-Mn(2) (158.96)	AFM	
		Mn(1)-O(3)-Mn(2) (152.32)	FM	
BiMnO_3.08_	*P2*_1_*/c*	Mn(1)-O(1)-Mn(2) (144.08)	AFM	Mn(1)-O(1): 2.045(7); Mn(1)-O(2): 1.983(5); Mn(1)-O(3): 1.922(6); Mn(1)-O(4): 2.098(6); Mn(1)-O(5): 1.993(6); Mn(1)-O(6): 1.944(5); Mn(2)-O(1): 2.008(8); Mn(2)-O(3): 2.121(2); Mn(2)-O(5): 1.900(4); Mn(3)-O(2): 2.091(6); Mn(3)-O(4): 2.029(2); Mn(3)-O(6): 1.965(3);
		Mn(1)-O(2)-Mn(3) (160.10)	FM	
		Mn(1)-O(3)-Mn(2) (154.28)	FM	
		Mn(1)-O(4)-Mn(3) (157.91)	FM	
		Mn(1)-O(5)-Mn(2) (153.26)	FM	
		Mn(1)-O(6)-Mn(3) (163.87)	AFM	
BiMnO_3.14_	*Pnma*	Mn-O(1)-Mn (155.87)	AFM	Mn-O(1): 1.995(3); Mn-O(2): 1.935(2); Mn-O(2): 2.027(7).
		Mn-O(2)-Mn (172.38)	FM	

Coordinates of the Mn ions at 300 K calculated for BiMnO_3.02_ (SG #15, *C2/c*): Mn(1)—*4e* (0, 0.216, 0.75); Mn(2)—*4d* (0.25, 0.25, 0.5); for BiMnO_3.08_ (SG #14, *P2*_1_*/c*): Mn(1)—*4e* (0.271, 0, 0.22); Mn(2)—*2b* (0.5, 0, 0); Mn(3)—*2c* (0, 0, 0.5); for BiMnO_3.14_ (SG #62, *Pnma*): Mn—*4b* (0; 0; 0.5). The type of exchange interaction (FM or AFM) is noted for each ternary Mn-O-Mn.

## Data Availability

Not applicable.
